# Intracranial pressure dynamics, cerebral autoregulation, and brain perfusion after decompressive craniectomy in malignant middle cerebral artery infarction: is there a role for invasive monitoring?

**DOI:** 10.1007/s00701-025-06537-0

**Published:** 2025-05-09

**Authors:** Modar Alhamdan, Anders Hånell, Timothy Howells, Anders Lewén, Per Enblad, Teodor Svedung Wettervik

**Affiliations:** Department of Medical Sciences, Section of Neurosurgery, Uppsala University, SE-751 85 Uppsala, Sweden

**Keywords:** Cerebral autoregulation, Decompressive craniectomy, Intracranial pressure, Malignant media infarction, Neurointensive care, Pressure reactivity index

## Abstract

**Objective:**

Malignant middle cerebral artery infarction (MMI) is a severe neurological condition. Decompressive craniectomy (DC) is an established lifesaving surgical treatment. However, the role of neurocritical care with monitoring and management of the intracranial pressure (ICP), pressure reactivity index (PRx), cerebral perfusion pressure (CPP), and optimal perfusion pressure (CPPopt) remain unclear. This study aims to examine the dynamics of these variables post-DC in relation to clinical outcome.

**Methods:**

This retrospective study included 70 MMI patients who underwent DC with ICP monitoring of at least 12 hours and available data of clinical outcome (modified Rankin Scale [mRS] at 6 months). The associations between mRS and cerebral physiology (ICP, PRx, CPP, and ∆CPPopt) was analysed and presented in different outcome heatmaps over the first 7 days following DC.

**Results:**

ICP above 15 mmHg was associated with unfavourable outcome, particularly for longer durations. As PRx exceeded zero, outcome worsened progressively, and values above 0.5 correlated to poor outcome regardless of duration. As CPP dropped below 80 mmHg, there was a transition from favourable to unfavourable outcome. Negative ∆CPPopt, particularly below -20 mmHg, corresponded to unfavourable outcome. In two-variable heatmaps, elevated PRx combined with high ICP, low CPP or negative ∆CPPopt correlated with worse outcome.

**Conclusion:**

Invasive ICP-monitoring may provide prognostic information for long-term recovery in MMI patients post-DC. The study highlighted disease-specific optimal physiological intervals for ICP, PRx, CPP, and ΔCPPopt. Of particular interest, the autoregulatory variable, PRx, influenced the safe and dangerous ICP, CPP, and ∆CPPopt intervals.

**Supplementary Information:**

The online version contains supplementary material available at 10.1007/s00701-025-06537-0.

## Introduction

Malignant middle cerebral artery (MCA) infarction (MMI) constitutes almost 10% [32] of all ischaemic stroke cases and is characterised by severe cerebral ischaemia [13]. Cytotoxic and vasogenic brain oedema develops within 2 to 5 days from onset and may lead to elevated intracranial pressure (ICP), reduced consciousness, and brain herniation [10]. Untreated, the mortality rate is 40–80% [7, 44]. Decompressive hemicraniectomy (DC) is currently the main treatment strategy to alleviate the brain herniation in selected cases [13, 44]. Several randomised controlled trials (RCTs) suggest that DC in MMI effectively reduces mortality and increases the rate of functional recovery, particularly in younger adults [23].

In general, after DC, the patients are treated in a neurointensive care (NIC) unit for the purpose of optimising brain physiology, saving vulnerable penumbra, and avoiding secondary brain injury. However, the level of evidence is weak regarding optimal treatment targets and the role for invasive monitoring of brain physiology, such as ICP [17, 45]. In other severe acute brain injuries, such as traumatic brain injury (TBI) and aneurysmal subarachnoid haemorrhage (aSAH) [36, 42], multimodality monitoring is considered fundamental during NIC. Most likely, the limited interest in monitoring brain physiology post-DC in MMI is related to DC being last-tier treatment of intracranial hypertension and low ICP postoperatively [14, 24, 27].

Furthermore, ICP may be useful, together with arterial blood pressure (ABP), to indicate the cerebral perfusion pressure (CPP) as a surrogate measure of the cerebral blood flow (CBF). In addition, ICP and ABP may provide information about the cerebral pressure autoregulatory function (pressure reactivity index [PRx]) of the brain [5, 47, 48]. PRx is the moving 5-min correlation coefficient between ICP and ABP. Higher values indicate worse cerebral autoregulation and are associated with unfavourable outcome in TBI and aSAH [5, 33, 41]. PRx varies with CPP in a U-shaped way and the CPP with the concurrently lowest PRx has been ascribed the CPP level where pressure autoregulation works best, i.e. optimal (CPPopt) target [2, 31]. Keeping CPP close to CPPopt has been associated with better brain tissue oxygenation [16], energy metabolism [34], and outcome [2, 31] in TBI, but less is known in other acute brain injuries [35]. Thus, it is possible that the individualised and temporally dynamic CPPopt may be an even better CBF surrogate target compared to CPP for all patients.

Although a severe primary brain injury has already occurred in post-DC MMI patients, it may still be important to save viable penumbra [28]. MMI patients often suffer from pre-existing chronic arterial hypertension and cerebrovascular diseases [1]. These patients may exhibit persisting cerebrovascular occlusion from the initial thrombus/embolus and could develop elevated ICP due to brain oedema to various extents [13]. Therefore, it is possible that they could benefit postoperatively from individualised therapy based on ICP, PRx, and CPP/CPPopt monitoring data. However, currently, there is only a handful of studies that are mainly focused on ICP-monitoring in MMI and there is a need for larger studies of high-frequency physiological data.

Thus, the main aim of this study was to determine the dynamics of ICP, PRx, CPP, and CPPopt over the first 7 days post-DC in MMI in relation to functional outcome. We also aimed to investigate the potential interaction between the cerebral autoregulatory status (PRx) with ICP, CPP, and CPPopt.

## Materials and methods

### Patients and study design

This observational, single-centre study was conducted at the Department of Neurosurgery, Uppsala University Hospital, Uppsala, Sweden. The Department provides neurosurgical care to approximately two million people living in the Uppsala County and seven near-by counties with local hospitals. MMI patients who were eligible for DC surgery, with impaired consciousness or at risk of deterioration, were transported to the NIC or neuro-intermediate care unit in Uppsala. Patients above approximately 70 years of age and with significant co-morbidities were not considered for DC and therefore not admitted. Uppsala is also a referral centre for neurointerventional thrombectomy and when revascularization could not be achieved, those patients were also potential candidates for DC. One hundred and one patients were treated with unilateral DC due to MMI at our centre over a period of 15 years (2008–2022). After excluding 11 patients without ICP-monitoring, 14 patients with less than 12 h of ICP data, and 6 patients without long-term clinical outcome data, the final study cohort comprised 70 patients.

### Management protocol

DC surgery was indicated in patients with significant radiological mass effect (midline shift > 5 mm) and reduced consciousness (Glasgow Coma Scale (GCS) ≤ 12). While the indication was not strictly based on predefined trial criteria, it was generally in line with the clinical definitions of malignant infarction used in previous RCTs, i.e. typically at least two-thirds of the MCA territory [44]. We required focal mass effect to avoid operating patients with only lesional symptoms. Relatively alert (GCS of 9–12) patients before surgery were generally extubated immediately postoperatively and did not receive an ICP monitor. Preoperatively unconscious patients (GCS ≤ 8) or patients with anticipated prolonged recovery were postoperatively kept intubated, mechanically ventilated, and received invasive ICP-monitoring. A parenchymal pressure device (Codman ICP Micro-Sensor, Codman & Shurtleff, Raynham, MA) was generally used for ICP monitoring in these cases. ICP was kept below 20 mmHg, pO_2_ above 12 kPa, pCO₂ between 4.5–5.5 kPa, and arterial glucose between 5 and 10 mmol/L. Target CPP was above 60 mmHg, which served as a lower threshold, but no general upper threshold was applied, although values above 100 mmHg were generally avoided. Normovolemia, normonatremia, and normothermia were also targeted. Repeated CT scans were performed even with normal ICP to exclude herniation of the brain through the craniectomy with awareness that this may occur without ICP increase in decompressed patients. The head of the bed was elevated to 30°. The patients were sedated with intravenous propofol infusion and received morphine for analgesia. Neurological wake-up tests were done 3–6 times per day, but patients were kept sedated if ICP was high. In case of intracranial hypertension and/or herniation due to hydrocephalus development, cerebrospinal fluid (CSF) was drained via an inserted ventricular catheter against a pressure level of around 15 mmHg. Thiopental infusion was only used in rare cases of severe intracranial hypertension. Intravenous fluids at first hand and inotropes or vasopressors (dobutamine or norepinephrine) at second hand, were used to maintain CPP above the lower limit.

### Clinical variables and outcome

Data on demography (age, sex), usage of antithrombotic agents pre-stroke, admission status (GCS motor score, hemiparesis, and dysphasia), treatments (thrombolysis, thrombectomy), clinical status before DC (GCS M and pupillary status), imaging before (infarction volume, midline shift, and level of basal cistern compression) and after DC (midline shift and level of basal cistern compression), and timing of DC in relation to the onset of stroke symptoms. The infarction volume was measured with the BrainLab software (Germany Headquarters, Munich, Germany) based on the delimitable infarction area on CT imaging before DC by one of the authors (TSW).

Modified Rankin Scale (mRS) was assessed at follow-up around 6 months post-DC based on medical records. mRS extends from 0 (no symptoms) to 6 (death) [4]. Favourable vs. unfavourable outcome was defined as mRS 0 to 3 vs. mRS 4 to 6.

### Data collection and analyses

ICP data were collected from the monitoring devices. Arterial blood pressure (ABP) was monitored via an arterial line at heart level. All physiological variables were collected at 100 Hz into the Odin software [15]. CPP was calculated as the difference between mean arterial blood pressure (MAP) and ICP [30]. PRx was defined as the moving 5-min window of Pearson’s correlation between 10 s-values of ICP and ABP [5, 47, 48]. CPPopt was calculated as the CPP with the lowest PRx over the last 4 h. [2] ∆CPPopt was calculated as CPP – CPPopt. The CPPopt yield was 54%. The physiological variables were analysed over the first seven days post-DC. For descriptive purposes, the median values of ICP, PRx, CPP, and CPPopt were calculated. The percentage of good monitoring time (%GMT) of ICP > 10 and > 20 mmHg were calculated, to indicate the burden of ICP-insults. The threshold at 20 mmHg was chosen in accordance with our treatment protocol and the 10 mmHg-threshold was chosen since post-DC patients usually have relatively low ICP and the outcome threshold may be lower than for patients with an intact neurocranium. The %GMT of PRx > 0.0 and + 0.20 was also analysed. PRx above zero indicates an early transition zone from pressure reactive to passive cerebral vessels, while + 0.20 has been suggested to indicate the limit of autoregulation in other acute brain injuries [3]. The %GMT of CPP < 60 mmHg and 80 mmHg was also analysed, as 60 mmHg is consistent with our treatment protocol and 80 mmHg was explored as we hypothesised that these patients may exhibit a right-sided autoregulatory plateau due to co-morbidities such as hypertension. We also explored %GMT of ΔCPPopt ± 5 and 10 mmHg, as ± 5 mmHg is consistent with the phase II-trial (COGiTATE) [43] on CPPopt in TBI and ± 10 mmHg was considered as a “broad range”.

### Visualisation methods

First, we explored the association between demographic (age), clinical (GCS M before DC), and radiological (midline shift after DC) risk factors with cerebral physiology (median values of ICP, PRx, CPP, and ∆CPPopt over the first 7 days). The correlation coefficients were calculated using Spearman analysis. Each coefficient (grid cell) was expressed as a colour employing a colour scale over the range −0.3 to + 0.3 (red to blue).

Second, we explored the association between cerebral physiology (ICP, CPP, ∆CPPopt, and PRx) and outcome (mRS) using an adapted version of the Guiza method [8], with a custom-written R-script, which has been previously developed by one of the authors (AH) [37–39, 41]. The first analysis investigated the combined effect of insult intensity and duration over the first 7 days post-DC for each variable in relation to mRS [38]. The ICP spanned from 5 to 30 mmHg and PRx from −1 to + 1. Since both too low and too high CPP/ΔCPPopt might be dangerous, insults below and above certain thresholds were analysed separately. The above threshold of CPP extended from 70 to 100 mmHg, while the below threshold was 40–70 mmHg. Similarly, the ∆CPPopt above threshold spanned from 0 to + 30, whereas the below threshold ranged from −30 to 0 mmHg. The resolution was 1 mmHg per grid cell for ICP, CPP, and ∆CPPopt, and 0.1 per grid cell for PRx. The duration ranged from 0 to 120 min (3 min per grid cell). The number of insults per grid cell, e.g., for ICP above 15 mmHg for 30–32 min (3 min/grid cell), was counted for every patient, and then divided by the GMT of the patient, to adjust for potential differences in the amount of monitoring data and then correlated with mRS. Positive correlation coefficients indicated an association between higher number of insults and poor outcome, and vice versa for the negative correlation. To produce smoother images, each grid cell was divided into 3 * 3 sub cells, followed by application of a Gaussian kernel filter (standard deviation of 2 grid cells). The final correlation values were visualised using the jet colour scale where blue indicates favourable and red unfavourable outcome. Grid cells with less than 20 patients with such an episode were coloured as white. Complementary density heatmaps were created by counting the number of observations within each grid cell and dividing it by the highest count among all grid cells. Since short insults were much more prevalent than longer insults, the logarithmic density was found to be more informative than the actual density and was therefore used. A similar smoothing process was done as described above. Frequent episodes were coloured as red and rare episodes as blue.

Furthermore, outcome (mRS) was analysed in relation (Spearman) to the %GMT within certain cerebral physiological intervals of single-variables, as a summary measure over the first 7 days post-DC as well as being subdivided into 21 eight-hour intervals (7 days) to assess any potential temporal dynamics [41]. These plots were divided into multiple, separate grid cells for each cerebral physiological variable; ICP (range 0–30 mmHg, 30 grid cells, 1 mmHg per grid cell), PRx (range −1.00 to + 1.00, 20 grid cells, 0.10 coefficient interval/grid cell), CPP (range 40 to 100 mmHg, 30 grid cells, 2 mmHg/grid cell), and ΔCPPopt (range −30 to + 30 mmHg, 30 grid cells, 2 mmHg/grid cell). In the summary plots, the %GMT was calculated within each interval/cell over the first 7 days post-DC. In the temporally divided plot, the same analyses were conducted over 21 eight-hour intervals. Furthermore, corresponding two-variable heatmaps were created to determine if the cerebral pressure autoregulatory status (PRx) interacted with ICP, CPP, or ΔCPPopt in relation to outcome [37, 39]. These plots were based on multiple grid cells for combinations of PRx with ICP, CPP, or ΔCPPopt, for the same ranges and intervals as in the single-variable analysis. Thus, the PRx/ICP plot included 600 cells (20 PRx intervals [range −1 to + 1, 0.1 per step] * 30 ICP intervals [range 0 to 30 mmHg, 1 mmHg per step]). Similarly, both the PRx/CPP and the PRx/ΔCPPopt plots included 600 grid cells. After setting the coordinates of these maps, the %GMT over the 7 days post-DC was calculated for each patient for every grid cell and correlated with mRS. This resulted in a single correlation value (Spearman) for each grid cell. To produce smoother images, each grid cell was divided into 3 * 3 sub cells followed by application of a Gaussian kernel filter (standard deviation of 2 grid cells). The final correlation values were visualised using the jet colour scale (blue = favourable and red = unfavourable). The colour scale was limited to correlations within ± 0.30 and results from grid cells with less than 5 patients that had at least 5 min of monitoring time were coloured as white. In addition, complementary data density heatmaps were created by counting the number of observations within each grid cell and dividing it by the highest count among all grid cells. A similar smoothing process was done as described above. Frequent episodes were coloured as red and rare episodes as blue.

### Statistical analyses

Continuous/ordinal variables were described as medians (first quartile – third quartile) and categorical variables as numbers (percentage). The Spearman rank test was used to explore the correlations between cerebral physiology and demography, clinical variables, radiology, and clinical outcome. A *p*-value below 0.05 was considered statistically significant. RStudio (version 2024.09.0) was used for the statistical analyses.

## Results

### Demography, injury severity, treatments, and outcome

In this cohort of 70 MMI patients treated with DC (Table 1), the median age was 57 (51–62) years and 79% were male. Pre-stroke, 10% of patients used anticoagulants and 17% antiplatelets. At admission, all patients were hemiparetic and 41% exhibited dysphasia. Thirty percent had been treated with thrombolysis and 10% had undergone mechanical thrombectomy. The median time from onset of stroke to DC was 41 (29–60) hours. Before DC, the median GCS M was 5 (5–6), the median midline shift was 11 (8–13) mm, and the median infarct volume was 254 (200–303) cm^3^. At follow-up, the median mRS was 4 (4–4), 20% had recovered favourably, and 7% were deceased. Two patients (3%) developed postoperative haemorrhage exceeding 10 mL, one of which was intraparenchymal and one epidural.

**Table 1:** Demographics, injury characteristics, admission status, treatments, and outcome

Patients, *n*	70
Age (years), median (Q1–Q3)	57 (51–62)
Sex (male), *n* (%)	55 (79%)
Pre-stroke anticoagulants, *n* (%)	7 (10%)
Pre-stroke antiplatelets, *n* (%)	12 (17%)
GCS M at admission (scale), median (Q1–Q3)	6 (5–6)
Hemiparesis at admission (yes), *n* (%)	70 (100%)
Dysphasia at admission (yes), *n* (%)	29 (41%)
Thrombolysis (yes), *n* (%)	21 (30%)
Thrombectomy (yes), *n* (%)	7 (10%)
GCS M before DC (scale), median (Q1–Q3)	5 (5–6)
Pupillary status before DC (normal/1 unreactive/2 unreactive), *n* (%)	58/12/0 (83/17/0%)
Midline shift before DC (mm), median (Q1–Q3)	11 (8–13)
Basal cisterns before DC (open/compressed/obliterated), *n* (%)	6/61/3 (9/87/4%)
Infarct volume (cm^3^), median (Q1–Q3)	254 (200–303)
Midline shift after DC (mm), median (Q1–Q3)	3 (1–6)
Basal cisterns after DC (open/compressed/obliterated), *n* (%)	59/9/2 (84/13/3%)
Time from onset of stroke to DC (hours), median (Q1–Q3)	41 (29–60)
mRS (scale), median (Q1–Q3)	4 (4–4)
Favourable outcome, *n* (%)	14 (20%)
Mortality, *n* (%)	5 (7%)

*Q1* First quartile; *Q3* Third quartile; *GCS M* Glasgow Coma Scale Motor score; *DC* Decompressive hemicraniectomy; *mRS* modified Rankin Scale. Favourable outcome = mRS ≤ 3, 6 months post DC. Mortality (at 6 months after DC)

### Cerebral physiology

#### Cerebral physiology the first seven days after DC

Descriptive data of the cerebral physiological variables the first 7 days post-DC are presented in Supplementary Table 1. In brief, the median ICP was 11 (9–13) mmHg, the median PRx was 0.14 (0.08–0.28), the median CPP was 79 (74–85) mmHg, and the median CPPopt was 78 (72–82) mmHg.

#### Cerebral physiology vs. demographic, clinical variables, and imaging

**Fig. 1:**
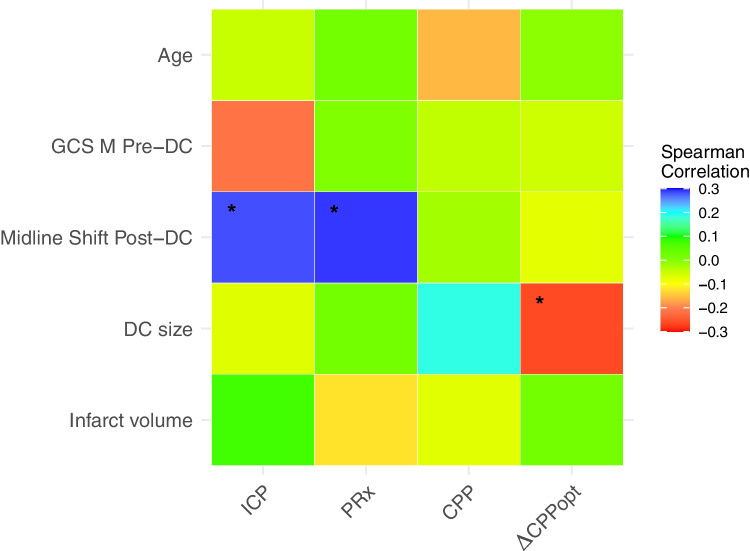
Cerebral physiological variables post-DC vs. predisposed and surgical factors. Grid heat map illustrating age (years), GCS M pre-DC (scale), midline shift post-DC (mm), DC size (cm^2^), and infarct volume (cm^3^) correlated to ICP, PRx, CPP, ∆CPPopt using Spearman analysis. Colours represent Spearman correlation coefficient. Blue refers to 0.3, and red to −0.3. No correlation coefficients were found to be outside this range. The symbol * refers to statistically significant *P*-value, below 0.05. As indicated, midline shift post-DC correlated significantly to higher ICP and PRx, while larger DC size correlated significantly to lower ∆CPPopt. GCS M = Glasgow Coma Scale Motor score. DC = decompressive hemicraniectomy. ICP = intracranial pressure. PRx = pressure reactivity index. CPP = cerebral perfusion pressure. ∆CPPopt = CPP – optimal CPP

Figure 1 and Supplementary Table 2 demonstrate the correlation (Spearman) between the medians of cerebral physiological variables and age, GCS M before DC, midline shift after DC, DC size, and infarct volume. As illustrated, these correlations were overall weak. However, greater midline shift after DC correlated significantly with higher ICP (r = 0.28, *p*-value = 0.02) and higher PRx (r = 0.29, *p* = 0.02), while larger DC size correlated with a lower ∆CPPopt (r = −0.26, *p* = 0.03).

#### Correlation between cerebral physiology and outcome

Cerebral physiological variables beyond certain thresholds were correlated to clinical outcome (mRS) using Spearman correlation analysis (Table 2). Higher %GMT of PRx > 0.0 correlated to higher mRS (r = 0.25, *p*-value = 0.04). There were non-significant associations between higher %GMT of ICP > 10 mmHg (r = 0.20, *p*-value = 0.095) and higher %GMT CPP < 80 mmHg (r = 0.16, *p*-value = 0.18) with higher mRS. ∆CPPopt showed a near-zero correlation (r = 0.092, *p*-value = 0.45) to mRS.

**Table 2:** Correlation between cerebral physiology and outcome

Variables	Spearman correlation	*P*-value
ICP		
Median value (mmHg)	0.21	0.079
ICP > 10 mmHg (%GMT)	0.20	0.095
ICP > 20 mmHg (%GMT)	0.17	0.15
PRx		
Median value (coefficient)	0.12	0.54
PRx > 0.0 (%GMT)	0.25	***0.037***
PRx > + 0.2 (%GMT)	0.23	0.053
CPP		
Median value (mmHg)	−0.18	0.13
CPP < 60 mmHg (%GMT)	0.15	0.21
CPP < 80 mmHg (%GMT)	0.16	0.18
ΔCPPopt		
Median value (mmHg)	−0.14	0.25
ΔCPPopt ± 5 mmHg (%GMT)	0.093	0.45
ΔCPPopt ± 10 mmHg (%GMT)	0.093	0.44

Spearman correlation between medians and values outside certain thresholds of NIC variables vs clinical outcome expressed in modified Rankin Scale. *ICP* Intracranial pressure; *PRx* Pressure reactivity index; *CPP* Cerebral perfusion pressure; *∆CPPopt* CPP – optimal CPP. *P*-value below 0.05 was considered statistically significant. *%GMT* Percentage of good monitoring time

#### Outcome visualisations of cerebral physiology – single-variable analyses

**Fig. 2:**
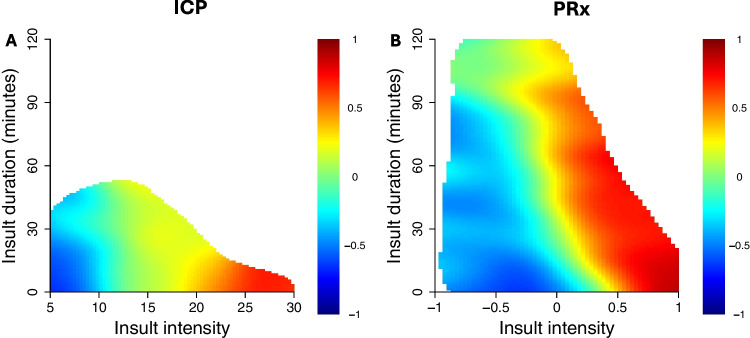
Insult duration and intensity of ICP and PRx in relation to clinical outcome. Figure A illustrates correlation between insult intensity and duration of ICP and mRS. The colour scale shows that red represents unfavourable outcome, blue favourable outcome, and white insufficient amount of data. ICP and PRx were analysed on above threshold basis. Figure B illustrates a similar plot for PRx. As indicated, there was a transition from better towards worse outcome for higher ICP and PRx for longer durations. ICP = intracranial pressure. PRx = pressure reactivity index. mRS = modified Rankin Scale

**Fig. 3:**
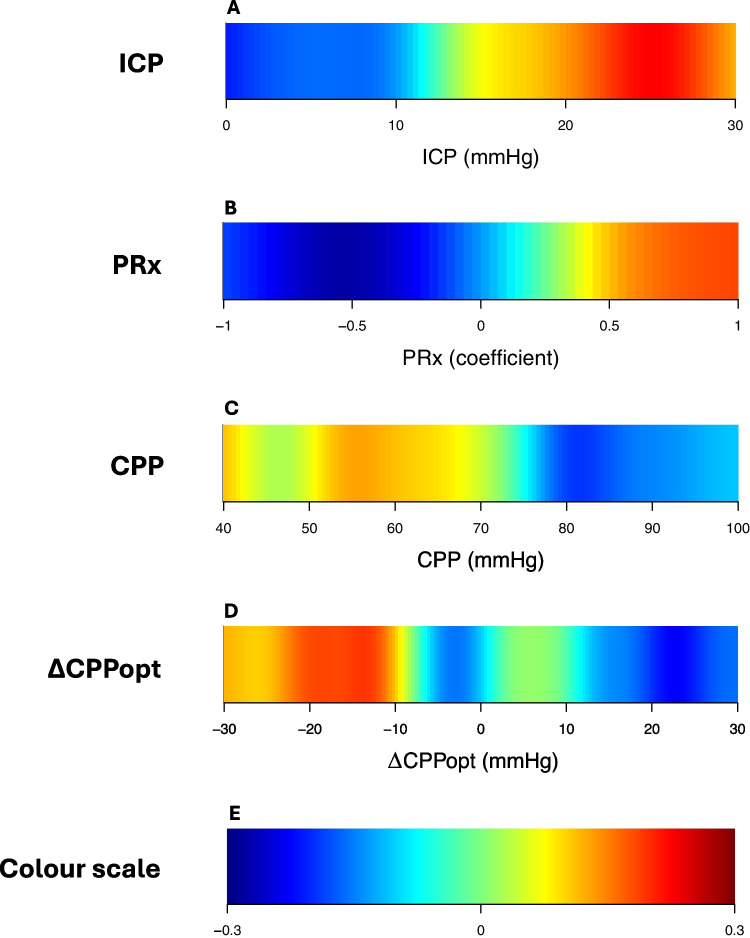
ICP, PRx, CPP, and ∆CPPopt in relation to clinical outcome. Figure A illustrates correlation between ICP level and mRS. The colour scale E indicates that red represents unfavourable outcome, and blue favourable outcome. The rest of the figures were created similarly, as figure B shows PRx, figure C CPP, and figure D ∆CPPopt. As the figures indicate, unfavourable outcome was associated with ICP above about 15 mmHg, PRx above around 0.5, CPP below about 70 mmHg, and ∆CPPopt below around −10 mmHg. ICP = intracranial pressure. PRx = pressure reactivity index. CPP = cerebral perfusion pressure. ∆CPPopt = CPP – optimal CPP. mRS = modified Rankin Scale

**Fig. 4:**
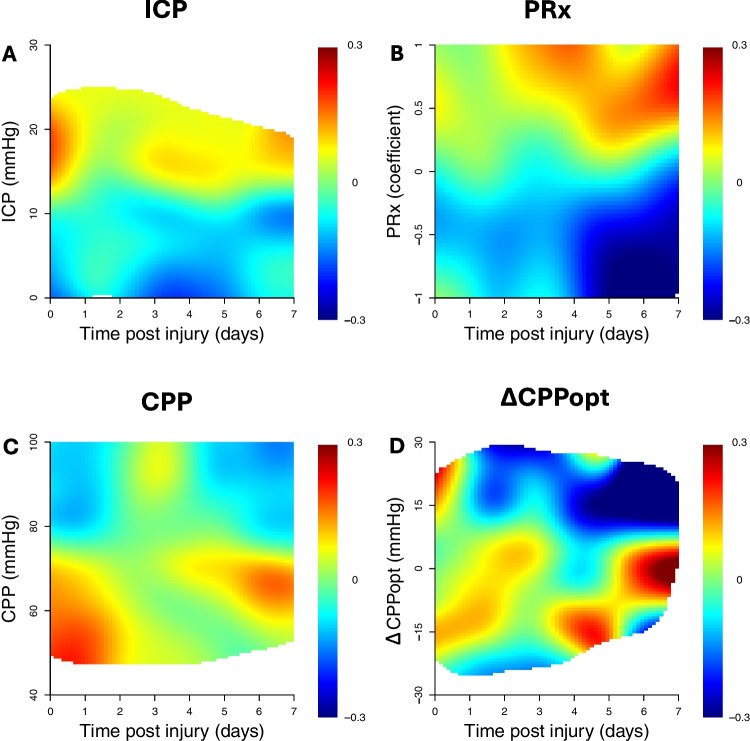
ICP, PRx, CPP, and ∆CPPopt the first 7 days post DC in relation to clinical outcome. Figure A presents correlation over the first seven days after DC between ICP level and mRS. The colour scale indicates that red represents unfavourable outcome, blue favourable outcome, and white insufficient data amount. The rest of the figures were created similarly, as figure B presents PRx, figure C CPP, and figure D ∆CPPopt. As demonstrated, unfavourable outcome correlated to ICP over roughly 15 mmHg, PRx over around 0, particularly after day 2, CPP below about 70 mmHg, and ∆CPPopt below roughly 0, particularly towards the end of the observation period. ICP = intracranial pressure. PRx = pressure reactivity index. CPP = cerebral perfusion pressure. ∆CPPopt = CPP – optimal CPP. mRS = modified Rankin Scale. DC = decompressive hemicraniectomy

For ICP, there was a transition from better (blue colour) towards worse (red colour) outcome when ICP exceeded approximately 15 mmHg (Figs. 2, 3, and 4), particularly for longer durations (Fig. 2). The threshold around 15 mmHg appeared to be consistent over the 7 days post-DC (Fig. 4). However, the association with worse outcome was slightly stronger between higher ICP and worse outcome the first day post-DC. As indicated in the density heatmaps (Supplementary Figs. 3 and 4), ICP was mostly around 5–15 mmHg.

As PRx progressed higher than 0.0, there was a gradient from favourable to unfavourable outcome (Figs. 2, 3, and 4), which appeared to be consistent the first seven days following DC (Fig. 4). Starting on day three, positive PRx corresponded particularly to worse outcome (Fig. 4). PRx above 0.0 for longer durations correlated to poorer outcome, while PRx over 0.5 was associated with unfavourable outcome regardless of duration (Fig. 2). PRx mainly ranged between 0.0 and 0.5, according to the density heatmaps (Supplementary Figs. 3 and 4).

**Fig. 5:**
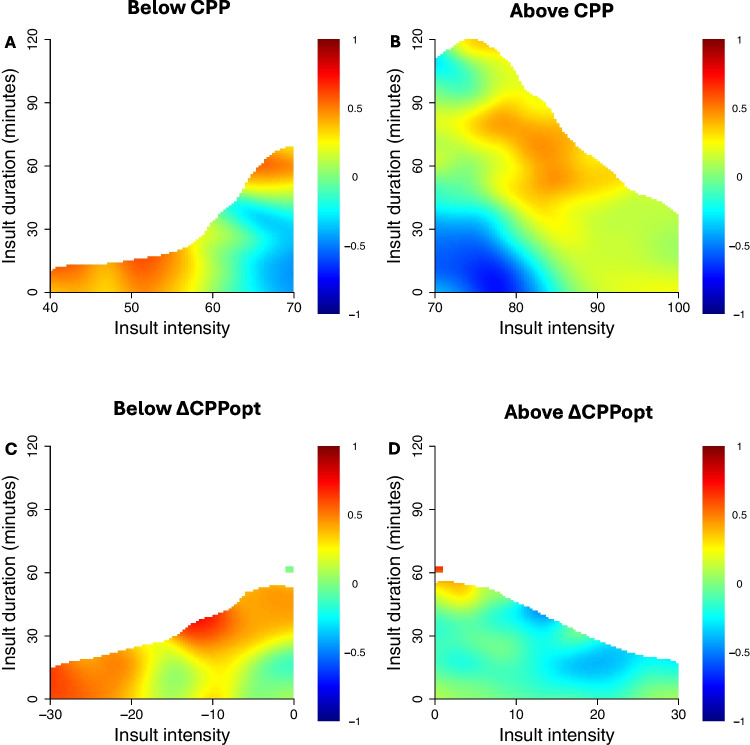
Insult duration and intensity of CPP and ∆CPPopt in relation to clinical outcome. CPP and ∆CPPopt were analysed on both above and below threshold basis. Figure A illustrates correlation between insult intensity and duration of below threshold CPP and mRS. The colour scale shows that red represents unfavourable outcome, blue favourable outcome, and white insufficient amount of data. The rest of the figures were composed similarly, as figure B shows above threshold CPP, figure C below threshold ∆CPPopt, and figure D above threshold ∆CPPopt. As indicated, unfavourable outcome correlated to CPP below 60 mmHg, below 70 mmHg for longer durations, and above 80 mmHg for longer durations. Also, unfavourable outcome was associated to ∆CPPopt below −20 mmHg, and below 0 mmHg for longer durations. CPP = cerebral perfusion pressure. ∆CPPopt = CPP – optimal CPP. mRS = modified Rankin Scale

Furthermore, there was a transition from favourable to unfavourable outcome when CPP dropped below 80 mmHg (Figs. 3, 4, and 5), which seemed relatively consistent over the seven days after DC (Fig. 4). Insults of CPP below 60 mmHg correlated to poor outcome regardless of duration, whereas insults above 80 mmHg were harmful if they occurred for longer durations (Fig. 5). Density heatmaps show that CPP was mostly around 70–80 mmHg (Supplementary Figs. 3 and 4).

ΔCPPopt above 10 mmHg corresponded to favourable outcome (Figs. 3, 4, and 5), which appeared relatively consistent over the first seven days following DC (Fig. 4). However, ∆CPPopt below 10 mmHg was particularly associated to worse outcome the last two days of the observation period (Fig. 4). Insults of ∆CPPopt under zero for longer durations correlated to bad outcome, while ∆CPPopt below −20 mmHg predicted unfavourable outcome regardless of duration (Fig. 5). As density heatmaps indicate, ∆CPPopt was approximately between −5 and 10 in most observations (Supplementary Figs. 3 and 4).

#### Outcome visualisations of cerebral physiology – the role of PRx in two-variable analyses

**Fig. 6:**
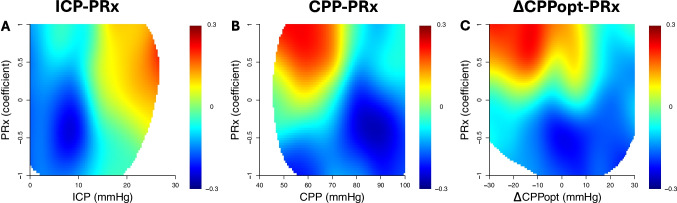
ICP, CPP, and ∆CPPopt combined with PRx in relation to clinical outcome. Figure A presents association of the combination of ICP-PRx with mRS 6 months after decompressive hemicraniectomy. The colour scale to the right illustrates that red represents unfavourable outcome, and blue favourable. White cells express insufficient amount of data. The rest of the heatmaps were created similarly as figure B presents CPP-PRx, and figure C ∆CPPopt-PRx. As demonstrated, high PRx combined with high ICP, low CPP, and low ∆CPPopt correlated particularly to worse outcome. ICP = intracranial pressure. PRx = pressure reactivity index. CPP = cerebral perfusion pressure. ∆CPPopt = difference between CPP and optimal CPP. mRS = modified Rankin Scale

In the two-variable heatmaps, the impact of the autoregulatory status on the association between ICP, CPP, and ΔCPPopt vs outcome was explored (Fig. 6). In the ICP/PRx heatmap, the combination of high ICP with high PRx was particularly associated with worse outcome, while the opposite held true for low ICP and low PRx. Furthermore, in the CPP/PRx heatmap, the combination of low CPP with high PRx was particularly associated with worse outcome, while high CPP and low PRx were favourable. Lastly, in the ΔCPPopt/PRx heatmap, the combination of PRx above zero with negative ΔCPPopt was particularly unfavourable.

## Discussion

This is the first study on the role of ICP, PRx, CPP, and ΔCPPopt based on in-depth, high-frequency monitoring analyses in MMI patients during the NIC course after DC surgery. In these patients, the physiological intervals associated with worse outcome occurred at a much lower ICP, above 15 mmHg, and higher CPP, below 80 mmHg, as compared to the applied ordinary management thresholds above 20 and below 60 mmHg, respectively. Furthermore, cerebral autoregulatory impairment with elevated PRx was common and significantly associated with worse outcome. In addition, PRx seemed to influence the safe intervals of ICP, CPP, and ΔCPPopt. Particularly, the combination of high PRx with high ICP, low CPP, and negative ΔCPPopt was more strongly correlated with worse patient outcome. Thus, these patients appear to be vulnerable to further ischaemia, and PRx may be useful to indicate when the lower limit of autoregulation is exceeded. Altogether, our study provides evidence that invasive ICP monitoring with corresponding data on CPP, PRx, and CPPopt may add value for clinical decision-making to optimise brain physiology and for predicting outcome in MMI patients post-DC. However, further studies based on larger, multi-centre cohorts are needed to validate our results.

Discussing the results more in detail, ICP insults above the traditional treatment threshold at 20 mmHg were very rare post-DC in our MMI patients. These findings were consistent with previous studies and expected, considering the dramatic increase in intracranial compliance following removal of a large part of the neurocranium [14, 18]. The postoperative ICP levels were also expected to be related both to extent of the underlying brain infarction causing secondary oedema and the size of the DC allowing the brain to swell externally [10]. The postoperative midline shift may be viewed as a combined surrogate measure of the extent of brain infarction/oedema and the surgical success to make a sufficiently large DC to alleviate the mass effect [17, 44, 45]. Postoperative ICP was significantly higher in the cases with greater postoperative midline shift, indicating that postoperative ICP levels may aid in the decision-making when to do an early postoperative CT scan to assess if the DC was sufficiently large [18, 22]. Interestingly, the correlation between postoperative ICP and area of DC was only weak and non-significant; a potential explanation could be that a smaller DC may sometimes be sufficient in relatively smaller infarctions. Furthermore, as outlined in the outcome heatmaps, there were clear transitions from better to worse patient recovery for higher ICP. Of note, the transitions from better towards worse outcome over the ICP range occurred at relatively lower values, around 15 mmHg, than the management protocol at 20 mmHg. It is possible that even moderately elevated ICP post-DC could contribute to further secondary brain injury due to cerebral venous compression against bone causing venous infarctions [26]. Also, higher ICP levels decrease CPP, which could drop below the lower limit of autoregulation [13]. This notion would support a lower ICP-threshold than the current level at 20 mmHg, which, in our case, is extrapolated from our TBI protocol [33, 34, 39, 42]. However, even if higher ICP, per se, may induce secondary injury, causality cannot be confirmed, and it is also highly related to the extent of underlying brain injury which by itself has a great impact on long-term outcome [10]. Furthermore, the association between ICP and mRS was weak and only reached marginal significance (*p* < 0.10). Altogether, our study indicates that postoperative ICP in DC-treated MMI patients carries prognostic information that may be of therapeutic significance and that the relevant threshold values may be lower than 20 mmHg.

Cerebral autoregulatory impairment with elevated PRx was frequent post-DC in our patients and was related to persistent radiological mass effect with greater midline shift on postoperative CT imaging. We speculate that there were both acute and chronic factors contributing to cerebral autoregulatory impairment. For the acute causes, many of these patients most likely still exhibited persistent occlusions in their proximal, ipsilateral cerebral vessels from the thrombus/emboli that caused the MMI [13]. The distal cerebral vessels may compensate for this to some extent by vasodilation to reduce the cerebrovascular resistance and augment CBF in watershed brain areas [13]. However, such brain areas remain vulnerable to CPP decreases, since it may transform a penumbral region to an infarction [12]. Moreover, it could be argued that PRx, as a global metric of cerebral autoregulation, may be insensitive to such subtle focal disturbances [12]. Nonetheless, PRx has provided valuable information in other conditions characterised by primarily focal brain injury, such as spontaneous intracerebral haemorrhage [19]. In addition, many patients with ischaemic stroke exhibit pre-existing cardiovascular diseases, such as arterial hypertension or cerebrovascular atherosclerosis [1]. Both of these acute and chronic factors could precipitate cerebral autoregulatory impairment, particularly causing a right-shifted autoregulatory curve making the patients more vulnerable to further ischaemic brain injury [35]. Of great interest, the cerebral autoregulatory status carried important prognostic information, as higher postoperative PRx was significantly associated with worse outcome. The transition from better to worse outcome appeared to occur around zero to + 0.5, particularly, when it occurred for episodes of longer durations, i.e., fairly consistent with other acute brain injuries such as TBI [33, 38, 39, 41]. Thus, our study provides novel evidence regarding the importance of PRx as an important monitoring variable of brain physiology in MMI, which appears consistent with previous studies on this variable in other acute brain injury conditions [29, 36, 38, 41, 46].

CPP insults below 60 mmHg were rare post-DC. There was no association between CPP-insults and age, severity of neurological injury, postoperative radiological outcome, or DC-area. This probably reflects that the patients were monitored and treated in our attentive NIC to keep ABP sufficiently high using fluids and, occasionally, inotropes/vasopressors to maintain the CPP target. Interestingly, there was a consistent trend towards worse outcome when CPP went below a relatively high CPP around 80 mmHg, although it did not reach statistical significance. This finding may be related to both the acute and chronic disturbances in cerebral autoregulation, as described above. In addition, we analysed the PRx-derived perfusion target, CPPopt, and found a trend towards worse outcome when ΔCPPopt became negative, providing some evidence for the role of CPPopt-targets in MMI. However, the CPPopt yield was relatively low in the post-DC state, which reduces the feasibility of using this target in this scenario. Furthermore, similar to the CPP-analyses, we found a trend towards worse outcome for greater burden of time outside ΔCPPopt-targets, but it did not reach statistical significance.

The two-variable outcome heatmaps clearly illustrated that the cerebral autoregulatory status (PRx) influenced the safe and dangerous ranges of the other cerebral physiological variables. Particularly, if cerebral autoregulation was impaired (high PRx), the tolerance for higher ICP, lower CPP, and negative ΔCPPopt was lower than if it was intact (low PRx). Interestingly, high CPP and positive ΔCPPopt seemed to be well-tolerated, even if PRx was high. These findings also support the notion that MMI patients are particularly vulnerable to ischaemic rather than hyperaemic injury [13]. However, extremely high CPP also impairs cerebral autoregulation and risks re-perfusion haemorrhage in ischaemic cerebral tissue [6, 25]. Altogether, these heatmaps indicate that PRx could aid in refining the ICP and CPP targets to alleviate the burden of secondary brain injury.

Cerebral autoregulation in MMI appears to be globally impaired but may be most critically affected in hypoperfused penumbral regions, making the identification of these focal areas essential for optimizing CPP-guided management [12]. In addition to autoregulatory assessment, downstream multimodal monitoring techniques may provide valuable insights, particularly when applied focally in penumbral tissue. These include monitoring of brain tissue oxygenation (pbtO₂), energy metabolism via microdialysis, and cortical spreading depolarisations through neurophysiological recordings [9, 11, 20, 40]. Compared to global invasive neuromonitoring, these approaches offer the advantage of detecting real-time focal pathophysiological disturbances [9, 11, 20, 37, 40]. However, data on their application following DC in MMI are currently limited [21], and further studies are needed to establish their clinical utility in this context.

Thus, our study provides further evidence about the value of invasive monitoring of brain physiology post-DC in MMI, in line with a recent study [18]. Furthermore, the ICP-derived variables of cerebral autoregulation and perfusion, PRx, CPP, and CPPopt, may also be very useful to calibrate NIC therapy to better avoid secondary, ischaemic brain injury. However, one important remark, while we found clear transitions of favourable and unfavourable cerebral physiological intervals, as compared to long-term functional outcome, these associations were overall weak. The main explanation for this is that all DC-treated MMI patients had a severe underlying brain injury that, per definition, necessitated such a last-tier intervention. It is obvious that this injury, affecting a major part of the MCA territory, contributing to hemiparesis and sometimes dysphasia [13], had the greatest impact on the long-term neurological sequalae. Still, our findings provide support that there is still some room for physiological optimisation that may be of value to reduce further secondary brain injury in penumbra and may in some cases save important functions such as the ability to walk or speak.

## Methodological considerations

The study had many strengths. It is the first study, based on a relatively large cohort of MMI patients, to investigate high-frequency cerebral physiology. This allowed to go beyond ICP-analyses and to explore the role of ICP-derived, autoregulatory metrics such as PRx and CPPopt, which have shown great promise to individualise NIC in TBI. Furthermore, we used advanced visualisation methods to allow for granular, in-depth analyses of transition zones in outcome, temporal dynamics, the combined effect of insult intensity and duration, and the interaction effect of PRx on ICP, CPP, and ΔCPPopt in relation to outcome.

The study also had some limitations. First, this was a retrospective, single-centre study, which limits external validity of our findings to some extent. Second, a subset of patients did not receive ICP-monitoring, which further limits the validity of our findings to the most severely injured patients who could not be immediately extubated. Third, PRx and CPPopt monitoring have been questioned as reliable indicators of cerebral physiology post-DC [49]. However, preliminary studies indicate that these variables still are reliable without an intact cranial vault [49]. Also, impaired cerebral autoregulation is heterogenous across the entire brain after MMI, and is likely most impaired in the infarct core and hypoperfused penumbra [12]. Since PRx is a global metric of cerebral autoregulation, it may not capture focal autoregulatory disturbances [12, 49]. However, studying the potential relevance of PRx in MMI is of great interest given the lack of prior studies on PRx in this condition.

## Conclusions

Invasive ICP-monitoring in MMI patients post-DC carried prognostic information for long-term recovery. While these patients rarely deviated from the management targets of ICP and CPP, the transition towards unfavourable outcome occurred at a lower ICP around 15 mmHg and higher CPP around 80 mmHg. Furthermore, cerebral autoregulatory impairment with elevated PRx was common and significantly associated with worse outcome. In addition, patients with a high %GMT exceeding the lower limit of cerebral autoregulation, as indicated with high PRx together with high ICP or low CPP, exhibited particularly worse patient outcome. Thus, PRx may be useful to determine the safe intervals of ICP and CPP. Also, there was a trend towards worse outcome for CPP below the PRx-derived CPPopt-target, however the overall yield was low which limits its potential value. Altogether, our study supports a potential prognostic and therapeutic role of ICP-monitoring and its derived perfusion and autoregulatory variables in MMI patients post-DC. Our study is novel in evaluating high-frequency physiological data from invasive neuromonitoring in this patient group. However, future studies based on larger cohorts are needed to validate our results.

## Supplementary Information

Below is the link to the electronic supplementary material.

Supplementary file1 (DOCX 1851 kb)

## Data Availability

Data are available upon reasonable request.

## Abbreviations

MMI: Malignant middle cerebral artery infarction
DC: Decompressive craniectomy
ICP: Intracranial pressure
PRx: Pressure reactivity index
CPP: Cerebral perfusion pressure
CPPopt: Optimal cerebral perfusion pressure
mRS: Modified Rankin Scale
MCA: Middle cerebral artery
RCTs: Randomized controlled trials
NIC: Neurointensive care
TBI: Traumatic brain injury
aSAH: Aneurysmal subarachnoid haemorrhage
ABP: Arterial blood pressure
CBF: Cerebral blood flow
MAP: Mean arterial pressure
CSF: Cerebrospinal fluid
GCS: Glasgow Coma Scale
GCS M: Glasgow Coma Scale Motor score
GMT: Good monitoring time
pO₂: Partial pressure of oxygen
pCO₂: Partial pressure of carbon dioxide
CT: Computed tomography
pbtO_2_: Brain tissue oxygenation pressure
