# Supplementary material for: Intracranial pressure dynamics, cerebral autoregulation, and brain perfusion after decompressive craniectomy in malignant middle cerebral artery infarction: is there a role for invasive monitoring?
Source: Acta Neurochir (Wien). 2025 May 9;167(1):135. doi: 10.1007/s00701-025-06537-0 (PMC12064602; doi:10.1007/s00701-025-06537-0)
Supplement: Supplementary file 1 — Supplementary file1 (DOCX 1851 kb) [file 701_2025_6537_MOESM1_ESM.docx]

# Supplementary tables and figures

**Supplementary table 1. Cerebral physiology the first 7 days after decompressive craniectomy**

| ICP (mmHg) | 11 (9–13) |
| --- | --- |
| ICP > 20 mmHg (%GMT) | 0.5 (0.1–1.6) |
| PRx (coefficient) | 0.14 (0.08–0.28) |
| PRx > 0.20 (%GMT) | 44 (35–58) |
| CPP (mmHg) | 79 (74–85) |
| CPP < 60 mmHg (%GMT) | 1 (0.5–4) |
| CPPopt (mmHg) | 78 (72–82) |
| ∆CPPopt (mmHg) | 2.0 (-0.75–4) |
| ΔCPPopt outside ±5 mmHg (%GMT) | 66 (61–72) |

Data is presented as median (Q1-Q3). Q1 = first quartile. Q3 = third quartile. ICP = intracranial pressure. PRx = pressure reactivity index. CPP = cerebral perfusion pressure. ∆CPPopt = CPP – optimal CPP.

**Supplementary table 2. Cerebral physiological variables post-DC vs. predisposed and surgical factors**

| Variables | | Spearman Correlation | P-value |
| --- | --- | --- | --- |
| Age (years) | ICP (mmHg) | -0.05 | 0.67 |
|  | PRx (coefficient) | 0.02 | 0.89 |
|  | CPP (mmHg) | -0.16 | 0.19 |
|  | ∆CPPopt (mmHg) | -0.01 | 0.95 |
| GCS M before DC (scale) | ICP (mmHg) | -0.22 | 0.07 |
|  | PRx (coefficient) | -0.00 | 1.00 |
|  | CPP (mmHg) | -0.05 | 0.71 |
|  | ∆CPPopt (mmHg) | -0.06 | 0.64 |
| Midline shift after DC (mm) | ICP (mmHg) | 0.28 | ***0.02*** |
|  | PRx (coefficient) | 0.29 | ***0.02*** |
|  | CPP (mmHg) | -0.02 | 0.85 |
|  | ∆CPPopt (mmHg) | -0.08 | 0.53 |
| DC size (cm^2^) | ICP (mmHg) | -0.07 | 0.56 |
|  | PRx (coefficient) | 0.02 | 0.88 |
|  | CPP (mmHg) | 0.19 | 0.12 |
|  | ∆CPPopt (mmHg) | -0.26 | ***0.03*** |
| Infarct volume (cm^3^) | ICP (mmHg) | 0.07 | 0.55 |
|  | PRx (coefficient) | -0.12 | 0.31 |
|  | CPP (mmHg) | -0.07 | 0.55 |
|  | ∆CPPopt (mmHg) | 0.02 | 0.89 |

GCS = Glasgow Coma Scale. DC = decompressive hemicraniectomy. ICP = intracranial pressure. PRx = pressure reactivity index. CPP = cerebral perfusion pressure. ∆CPPopt = CPP – optimal CPP. P-value of less than 0.05 was considered statistically significant.

**Supplementary figure 1. Selection of study population**

All adult patients surgically treated by a hemicraniectomy due to malignant MCA infarction, between 2008-2022 in the NIC unit at Uppsala University Hospital

N=101

Excluded at baseline:

Patients with no neurointensive monitoring

n=11

Patients with neurointensive monitoring less than 12h

n=14

Patients with no outcome observations

n=6

Included patients

n=70

Supplementary figure 1 presents the inclusion and exclusion process of the study population. The number of eligible patients was 101. Patients with no neurointensive data (n=11), patients with neurointensive data less than 12 hours (n=14), and patients with no outcome observations (n=6) were excluded. Thus, the final cohort consisted of 70 patients.

**Supplementary figure 2. Density of insult duration and intensity of ICP and PRx**

Figure A presents density distribution of insult intensity and duration of ICP. The colour scale indicates that red expresses higher density, and blue lower density. Figure B is created similarly for PRx. As indicated, ICP was mostly about 5–20 mmHg and below 15 minutes, while PRx was mostly between -0.5 and +0.5. ICP = intracranial pressure. PRx = pressure reactivity index.

**Supplementary figure 3. Density of ICP, PRx, CPP, and ∆CPPopt**

Figure A presents density distribution of ICP. The colour scale E explains that red expresses higher density of observations, and blue lower density. The rest of the figures were created similarly, as figure B shows PRx, figure C CPP, and figure D ∆CPPopt. As the figures indicate, ICP was mostly around 5–15 mmHg, PRx about 0–0.5, CPP around 70–80 mmHg, and ∆CPPopt about -5 and +10 mmHg. ICP = intracranial pressure. PRx = pressure reactivity index. CPP = cerebral perfusion pressure. ∆CPPopt = CPP – optimal CPP. mRS = modified Rankin Scale.

**Supplementary figure 4. Density of ICP, PRx, CPP, and ∆CPPopt the first 7 days post DC**

Figure A illustrates density distribution of ICP over the first seven days following DC. The shown colour scale explains that red represents higher density, and blue lower density. The grey lines in the density graphs indicate the percentage of patients with observations at that timepoint. The rest of the figures were created similarly, as figure B presents PRx, figure C CPP, and figure D ∆CPPopt. As indicated, ICP was mostly around 5–15 mmHg, PRx about 0–0.5, CPP around 70–80 mmHg, and ∆CPPopt about 0–10 mmHg. ICP = intracranial pressure. PRx = pressure reactivity index. CPP = cerebral perfusion pressure. ∆CPPopt = CPP – optimal CPP. mRS = modified Rankin Scale. DC = decompressive hemicraniectomy.

**Supplementary figure 5. Density of insult duration and intensity of CPP and ∆CPPopt**

Figure A presents density distribution of insult intensity and duration of below threshold CPP. The following colour scale explains that red expresses higher density, and blue less density. The rest of the figures were composed in a similar manner, as figure B presents above threshold CPP, figure C below threshold ∆CPPopt, and figure D above threshold ∆CPPopt. As demonstrated, CPP was mostly above around 60 mmHg and ∆CPPopt was mostly between ±20 mmHg. CPP = cerebral perfusion pressure. ∆CPPopt = CPP – optimal CPP.

**Supplementary figure 6. Density heatmaps of ICP, CPP, and ∆CPPopt combined with PRx**

Figure A shows distribution of density of ICP and PRx. As explained by the following colour scale, red represents more density, and blue less density. Figure B presents density distribution of ICP with PRx, and C ∆CPPopt with PRx. As indicated, ICP was mostly between around 5–15 mmHg, PRx about 0–0.5, CPP around 70–80 mmHg, and ∆CPPopt between about -5 and +10 mmHg. ICP = intracranial pressure. PRx = pressure reactivity index. CPP = cerebral perfusion pressure. ∆CPPopt = CPP – optimal CPP.
